# Circulating glutamine/glutamate ratio is closely associated with type 2 diabetes and its associated complications

**DOI:** 10.3389/fendo.2024.1422674

**Published:** 2024-07-18

**Authors:** Fei Han, Chaofei Xu, Xinxin Hangfu, Yanjie Liu, Yi Zhang, Bei Sun, Liming Chen

**Affiliations:** NHC Key Laboratory of Hormones and Development, Tianjin Key Laboratory of Metabolic Diseases, Chu Hsien-I Memorial Hospital and Tianjin Institute of Endocrinology, Tianjin Medical University, Tianjin, China

**Keywords:** diabetes, diabetic complications, metabolic syndrome, glutamine/glutamate ratio, serum amino acid profile

## Abstract

**Objective:**

This study aims to conduct a comprehensive investigation of the serum amino acid profiles of individuals with type 2 diabetes (T2D) and its related complications.

**Methods:**

Patients with T2D were enrolled in this study. Sixteen kinds of common amino acids in the fasting circulating were assessed through liquid chromatography-mass spectrometry (LC-MS). Subsequently, correlation, regression analyses, and receiver operating characteristic (ROC) curves were conducted to assess the associations between amino acids and clinical indicators.

**Results:**

Thirteen different kinds of amino acids were identified in diabetic patients, as compared with normal controls. The Glutamine/Glutamate (Gln/Glu) ratio was negatively correlated with BMI, HbA1c, serum uric acid, and the triglyceride-glucose (TyG) index, while it was positively correlated with HDL-C. Logistic regression analyses indicated that Gln/Glu was a consistent protective factor for both T2D (OR = 0.65, 95% CI 0.50-0.86) and obesity (OR = 0.79, 95% CI 0.66-0.96). The ROC curves demonstrated that Gln/Glu, proline, valine, and leucine provided effective predictions for diabetes risk, with Gln/Glu exhibiting the highest AUC [0.767 (0.678-0.856)]. In patients with T2D, Gln was the only amino acid that displayed a negative correlation with HbA1c (*r* = -0.228, *p* = 0.017). Furthermore, HOMA-β exhibited a negative correlation with Glu (*r* = -0.301, *p* = 0.003) but a positive correlation with Gln/Glu (*r* = 0.245, *p* = 0.017). Notably, logistic regression analyses revealed an inverse correlation of Gln/Glu with the risk of diabetic kidney disease (OR = 0.74, 95% CI 0.55-0.98) and a positive association with the risk of diabetic retinopathy (OR = 1.53, 95% CI 1.08-2.15).

**Conclusion:**

The Gln/Glu ratio exhibited a significant association with diabetes, common metabolic parameters, and diabetic complications. These findings shed light on the pivotal role of Gln metabolism in T2D and its associated complications.

## 1 Background

Diabetes affected over 500 million individuals in 2021 and significantly contributes to mortality, resulting in over 6 million deaths, with anticipated increases in the coming decades (1). These data highlight the critical necessity for effective disease prevention. Type 2 diabetes (T2D) represents 90% of all diabetes cases, with its prevalence on the rise due to sedentary lifestyles and increased adiposity (2). Moreover, T2D is associated with a diminished quality of life due to pain, limited mobility, anxiety, and depression (3). Various risk factors, including non-modifiable ones such as age and genetics, as well as modifiable factors like environment and lifestyle, have been linked to T2D. Factors like diet, body mass index (BMI), physical activity, alcohol consumption, and smoking have all shown links to the risk of developing diabetes (4).

Recently, significant efforts have been made to understand the pathogenesis of diabetes; however, there is considerable uncertainty in this area. There is a growing interest in the clinical application of metabolomics to identify a panel of biomarkers for detecting, predicting, and monitoring diabetes and its associated complications. Several studies have indicated a strong association between T2D, obesity, dyslipidemia, diabetes complications, and changes in metabolic signatures, particularly plasma amino acids (5–7). Despite this, the results of these studies have been conflicting.

Amino acid availability plays a crucial role in regulating intracellular signaling, hormonal secretion, and energy homeostasis. Branched-chain amino acids (BCAAs) like leucine, isoleucine, and valine are particularly noteworthy for their pivotal roles in controlling insulin secretion, glucose and lipid metabolism, central nervous system regulation of food intake, and energy balance (8, 9). The accelerated breakdown of tryptophan in the kynurenine pathway may contribute to the progressive decline in kidney function (10). Administering tryptophan prevents the development of non-alcoholic fatty liver disease in male Wistar rats (11). Additionally, levels of serum sulfur-containing amino acids have been linked to the risk of maternal gestational diabetes and adverse growth patterns in offspring (12). Lastly, amino acids like tryptophan, leucine, and valine have emerged as potential diagnostic markers for T2D due to the altered metabolism of these compounds in individuals with prediabetes and throughout the progression of T2D (13). Given the substantial association between diabetes and dietary habits, further investigation is essential to elucidate the intricate relationship between diabetes and amino acids.

Individuals with T2D are often afflicted by elevated peripheral neuropathy, cardiovascular diseases, and diabetic kidney disease (DKD) (14). Although numerous studies have investigated the relationship between amino acids and diabetes, the link to diabetic complications remains uncertain. A prior study suggested that serum BCAAs could be a reliable indicator of DKD (7). Analysis of blood metabolites through untargeted metabolome studies has indicated that L-arginine and threonic acid may potentially contribute to the development of non-proliferative diabetic retinopathy (DR) (15). Furthermore, glutamic acid metabolism has been implicated in the specific locus for diabetes-related coronary heart disease (16) and obesity (17).

The previous studies have underscored the significance of amino acids in diabetes; however, some of them merely compared amino acid profiles without delving into their association with diabetes (18, 19).

Additionally, T2D is often accompanied by metabolic dysfunctions such as obesity, dyslipidemia, and hyperuricemia. A prior study examined the link between amino acid profiles and diabetic-related parameters, including HOMA-IR, glycated hemoglobin (HbA1c), and dyslipidemia, but the study had a smaller number of enrolled patients (20). To date, there has been no systematic analysis in clinical studies regarding the relationship between amino acid profiles and diabetic complications.

This study aims to conduct a comprehensive investigation of the serum amino acid profiles of individuals with T2D and its related complications. Through the identification of stable biomarkers associated with the progression of T2D, we can deepen our understanding of the underlying mechanisms, potentially paving the way for novel insights and therapeutic targets for the prevention and management of T2D and its complications.

## 2 Materials and methods

### 2.1 Participants

A total of 110 patients with T2D who were admitted to the Chu Hsien-I Memorial Hospital Department of Tianjin Medical University participated in this study. Additionally, 42 age- and sex-matched volunteers from physical examination centers were included as the control group. The inclusion criteria comprised patients aged 18-80 years with a confirmed diagnosis of T2D as per previously documented criteria (21).

Exclusion criteria encompassed serious liver disease, rheumatism, cancer, infectious disease, nephropathy requiring dialysis, and other endocrine disorders (except diabetes mellitus). In addition, patients using drugs that affect glucose metabolism (except anti-diabetic drugs), such as glucocorticoids, were excluded. The clinical characteristics of all subjects are shown in **Table 1**. This study was approved by the Ethics Review Committee of Chu Hsien-I Memorial Hospital of Tianjin Medical University and in accordance with the Helsinki Declaration.

**Table 1:** Clinical characteristics of patients with T2D and the controls.

	NC (n=42)	T2D (n=110)	*P*-values
**Age (years)**	52.83 ± 10.70	56.14 ± 12.24	0.126
**Gender (female/male)**	10/32	39/71	0.170
**Smoking habits (no/yes)**	17/25	63/47	0.064
**Drinking habits (no/yes)**	29/13	70/40	0.531
**Diabetes duration (years)**	–	9.0 (2.75, 17.25)	–
**BMI (kg/m^2^)**	24.96 ± 4.19	27.68 ± 4.24 **	0.001
**HbA1c (%)**	5.51 ± 0.44	8.69 ± 2.12 ***	<0.001
**FPG (mmol/L)**	5.05 (4.6, 5.2)	7.7 (6.2, 9.5) ***	<0.001
**ALT (U/L)**	20.05 (15.4, 35.8)	22.55 (15.1, 36.8)	0.770
**AST (U/L)**	28.12 ± 11.12	26.51 ± 16.70	0.168
**γ-GGT (U/L)**	25.45 (16.3, 51.5)	29.95 (19.9, 46.2)	0.272
**CKD-EPI**	92.53 ± 12.93	89.46 ± 26.57	0.856
**SUA (μmol/L)**	399.87 ± 165.87	428.56 ± 120.75	0.070
**TG (mmol/L)**	1.53 (1.05, 2.47)	1.75 (1.23, 2.87)	0.232
**TC (mmol/L)**	5.27 ± 1.08	5.51 ± 1.55	0.767
**HDL-C (mmol/L)**	1.32 ± 0.31	1.11 ± 0.28 ***	<0.001
**LDL-C (mmol/L)**	3.43 ± 0.86	3.54 ± 1.02	0.972
**TyG**	1.04 ± 0.27	1.39 ± 0.47 ***	<0.001

*p < 0.05; **p < 0.01; ***p < 0.001.

### 2.2 Diagnostic criteria

Obesity was defined as BMI of at least 28 kg/m². The diagnosis of DKD, diabetic peripheral neuropathy (DPN) and, coronary heart disease (CHD) complied with the criteria reported previously (22–24). The presence of non-alcoholic fatty liver disease (NAFLD) was assessed by liver ultrasound imaging (FibroScan)—which is a noninvasive, simple and fast diagnostic method (25). All diabetic patients underwent both fundus photography and optical coherence tomography (OCT) examinations, with DR diagnosis and verification conducted by experienced ophthalmologists. Arteriosclerosis obliterans (ASO) was diagnosed through patient history and physical examination.

### 2.3 Laboratory examination

All participants fasted at 10:00 pm, after which venous blood was collected at 8:00 AM the following day. The blood samples were allowed to stand at room temperature for 30 minutes before being centrifuged at 3500 g for 15 minutes. Subsequently, the serum obtained after centrifugation was utilized to analyze various factors, encompassing blood glucose metabolism indicators such as fasting plasma glucose (FPG), fasting insulin, fasting C-peptide, and HbA1c; blood lipid metabolism indicators like high-density lipoprotein cholesterol (HDL-C), low-density lipoprotein cholesterol (LDL-C), triglycerides (TG), and total cholesterol (TC); liver function markers including alanine aminotransferase (ALT), aspartate aminotransferase (AST), and gamma-glutamyl transpeptidase (γ-GGT); and renal function parameters such as blood urea nitrogen (BUN), creatinine (Cr), the Chronic Kidney Disease Epidemiology Collaboration (CKD-EPI) formula, and uric acid (UA). The leftover serum samples were preserved at -80°C until the final analysis.

Amino acid levels in patient serum, including glutamine (Gln), glutamate (Glu), alanine (Ala), serine (Ser), proline (Pro), valine (Val), leucine (Leu), isoleucine (Ile), aspartic acid (Asp), lysine (Lys), methionine (Met), histidine (His), tryptophan (Tryp), phenylalanine (Phe), L-cystine (Cys), and L-threonine (Thre), were analyzed using liquid chromatography-mass spectrometry (LC-MS) in accordance with a previously published protocol (7). Initially, the serum sample was frozen and then combined with 5 mL of an internal standard mixture and 100 mL of a 1:1 blend of ethacrynic acid and methanol pre-cooled at -20°C. The shaker was pre-cooled to 4°C before vortexing the EP tubes for 5 minutes. Subsequently, the EP tubes were centrifuged at 14,000g for 15 minutes at 4°C. The supernatant from the EP tube was transferred to a new 1.5 mL enzyme-free EP tube and dried using nitrogen, with temporary storage at -80°C if not promptly analyzed. Redissolving the sample involved the addition of 100 mL of 20% methanol to the EP tube followed by vortexing for 2 minutes. The EP tube was then centrifuged at 4°C, 14,000g for 5 minutes. Lastly, the supernatant was carefully transferred to a sampling vial ensuring the absence of air bubbles, and then injected for LC-MS analysis.

Homeostatic Model Assessment for Insulin Resistance, HOMA-IR = FBG (mmol/L) × FINS (μU/mL)/22.5 (26).

Homeostatic Model Assessment for beta-cell function, HOMA-β = 20 × FINS (μU/mL)/(FPG (mmol/L) - 3.5) (26).

Triglyceride glucose (TyG) index = Ln (TG [mmol/L] × FBG [mmol/L]/2) (26).

### 2.4 Statistical analysis

All experimental data were sorted and analyzed using IBM SPSS version 20 (V20.0, IBM Corp, Chicago, USA). Prior to statistical analysis, the data were assessed for normal distribution and homogeneity of variance. Mean ± standard deviation was utilized for normally distributed data, while medians and interquartile ranges were used for non-normally distributed data, with nonparametric tests for comparison. Data were log10 transformed where necessary. Baseline characteristics and circulating amino acids were compared between normal controls (NC) and T2D cases using *t*-tests for continuous variables and chi-squared tests for categorical variables. Spearman’s rank correlation was employed to investigate the relationship between serum amino acid levels and clinical indicators in T2D patients. Univariate and multivariate logistic regressions were performed to assess the predictors. Both crude and adjusted odds ratios (OR) with 95% CI were calculated to determine the relationships between circulating amino acids, T2D, and other associated complications. Measures of diagnostic accuracy including the area under the curve (AUC), sensitivity, and specificity were computed. Receiver Operating Characteristic (ROC) curve analysis, covering a range from 0.5 to 1.0, was performed to assess the diagnostic value of amino acids. All analyses were two-tailed, with statistical significance set at *p* < 0.05.

## 3 Results

### 3.1 Clinical characteristics of patients with T2D and healthy controls

The table provided a comprehensive overview of the clinical characteristics of T2D patients and the control group. It revealed that factors such as age, gender, smoking and drinking habits, liver enzymes (ALT, AST, γ-GGT), kidney function (CKD-EPI), uric acid levels, TG, TC, and LDL-C did not exhibit statistically significant differences between the two groups (*p* > 0.05) (**Table 1**). However, the diabetes group showed higher levels of BMI, HbA1c, FPG, and TyG index, alongside reduced levels of HDL-C levels compared to the control group.

### 3.2 Concentrations of circulating amino acids in patients with T2D and healthy controls

**Figure 1:**
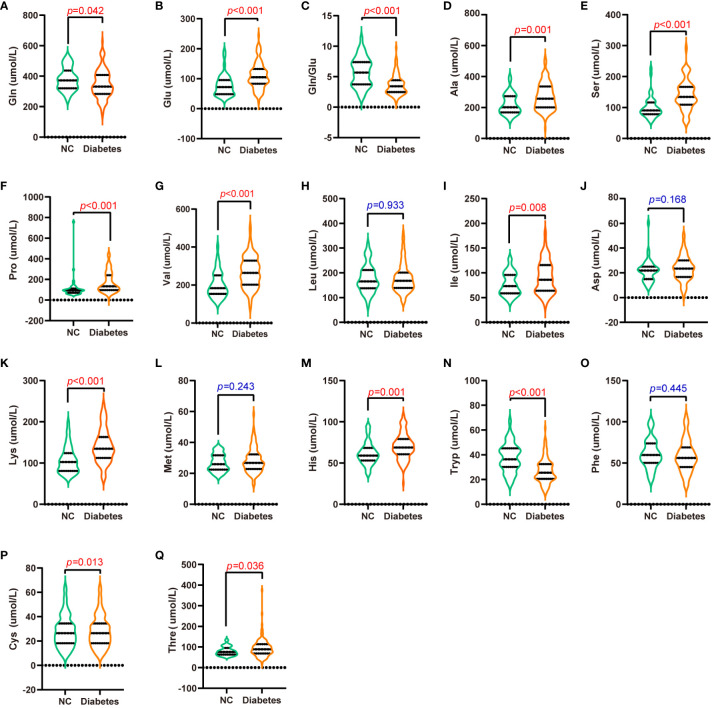
Serum amino acid levels in patients with T2D and normal controls. **(A-Q)** are Gln **(A)**, Glu **(B)**, Gln/Glu **(C)**, Ala **(D)**, Ser **(E)**, Pro **(F)**, Val **(G)**, Leu **(H)**, Ile **(I)**, Asp **(J)**, Lys **(K)**, Met **(L)**, His **(M)**, Tryp **(N)**, Phe **(O)**, Cys **(P)**, and Thre **(Q)** concentrations in the serum of patients with T2D and normal controls.

Amino acid profiles differed significantly between the normal and T2D groups (*p* < 0.05, **Figure 1**, **Supplementary Table S1**). Specifically, circulating Gln, Gln/Glu ratio, and Tryp were significantly decreased in the T2D group (*p* < 0.05). Conversely, the levels of Glu, Ala, Ser, Pro, Val, Ile, Lys, His, Cys, and Thre were significantly elevated in the T2D group (*p* < 0.05). On the other hand, Leu, Asp, Met, and Phe did not show statistically significant differences between the groups (*p* > 0.05).

### 3.3 Correlation analysis of circulating amino acids and metabolic indicators in T2D and healthy controls

**Figure 2:**
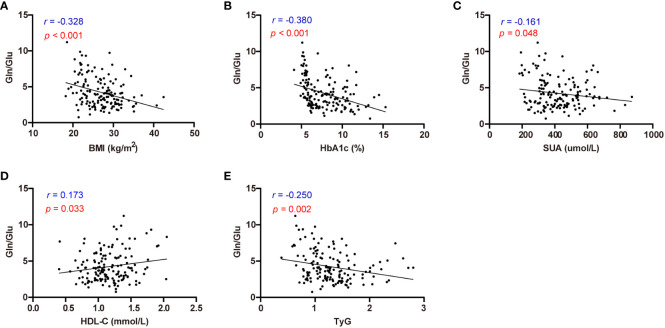
Correlation analysis of Gln/Glu ratio with BMI, HbA1c, SUA, HDL-C, and TyG index. **(A-E)** describes the correlations between Gln/Glu and BMI **(A)**, HbA1c **(B)**, SUA **(C)**, HDL-C **(D)**, and TyG index **(E)** respectively.

Correlation analysis was employed to investigate the relationship between circulating amino acid and various clinical parameters in individuals with T2D and healthy controls (**Figure 2**, **Supplementary Table S2**). BMI showed positive correlations with Glu, BCAAs, Lys, and Phe, while exhibiting negative correlations with Gln and Gln/Glu ratio (all *p* < 0.05). HbA1c showed positive correlations with Glu, Ala, Ser, Pro, BCAAs, and Lys, but unfavorable correlations with (all *p* < 0.05). Notably, Gln/Glu was the only parameter demonstrating a negative correlation with SUA concentration (*r* = -0.161, *p* = 0.048). TG exhibited positive correlations with BCAAs, Asp, and Phe (all *p* < 0.05), while TC demonstrated positive correlations with Asp (*r* = 0.210, *p* = 0.01). Furthermore, the Gln/Glu ratio was the only amino acid parameter showing a positive correlation with HDL-C (*r* = 0.173, *p* = 0.033). HDL-C showed negative correlations with Ala, Pro, BCAAs, Lys, Met, His, and Phe (all *p* < 0.05), whereas LDL-C exhibited a positive correlation with Asp (*r* = 0.162, *p* = 0.047). The TyG index, commonly used as a marker for assessing insulin resistance, exhibited a negative correlation solely with the Gln/Glu ratio (*r* = -0.250, *p* = 0.002). In addition, the TyG index showed positive correlations with BCAAs, Asp, and Phe (all *p* < 0.05). These findings suggest that the Gln/Glu ratio may be among the amino acids most commonly associated with metabolic parameters.

### 3.4 Multivariable logistic regression analysis of circulating amino acids and common metabolic syndrome

**Figure 3:**
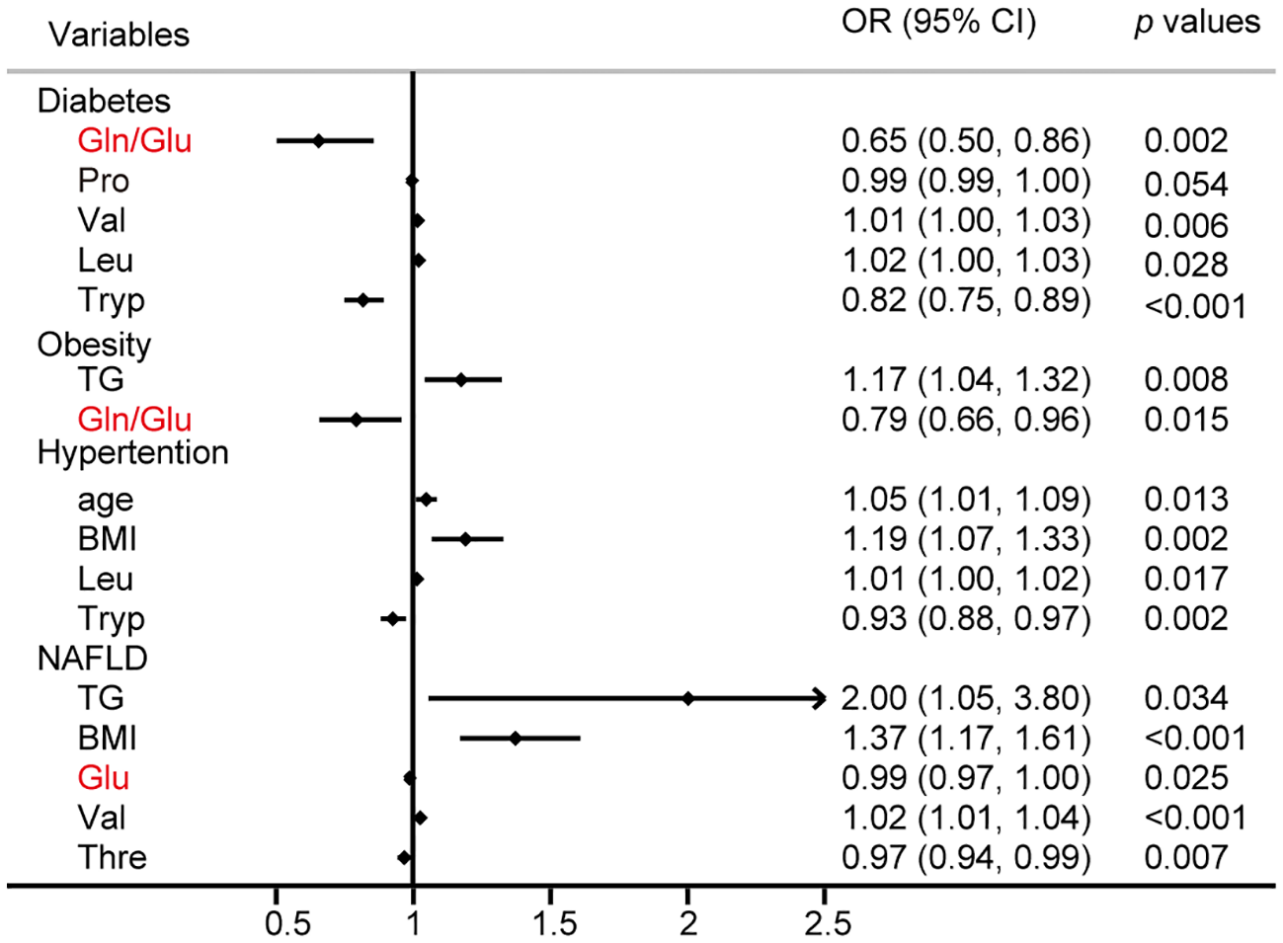
Logistic regression analysis for the risk factors of diabetes, obesity, hypertention, and NAFLD.

Logistic regression analysis was performed to identify the risk factors associated with the common metabolic syndrome, including T2D, obesity, hypertension, and NAFLD, respectively. The results of this study identified Gln/Glu ratio, Pro, Val, Leu, and Tryp as independent risk factors for diabetes, whereas TG and the Gln/Glu ratio emerged as independent risk factors for obesity. Additionally, age, BMI, Leu, and Tryp were identified as independent risk factors for hypertension. Notably, 29.2% of adults in the general population in China are affected by NAFLD (27), a condition closely associated with obesity, T2D, hyperlipidemia, and hypertension (28). The results showed that TG, BMI, Glu, Val, and Thre were all independent risk factors for NAFLD (**Figure 3**).

### 3.5 Evaluation of the circulating amino acids affecting diabetes

**Figure 4:**
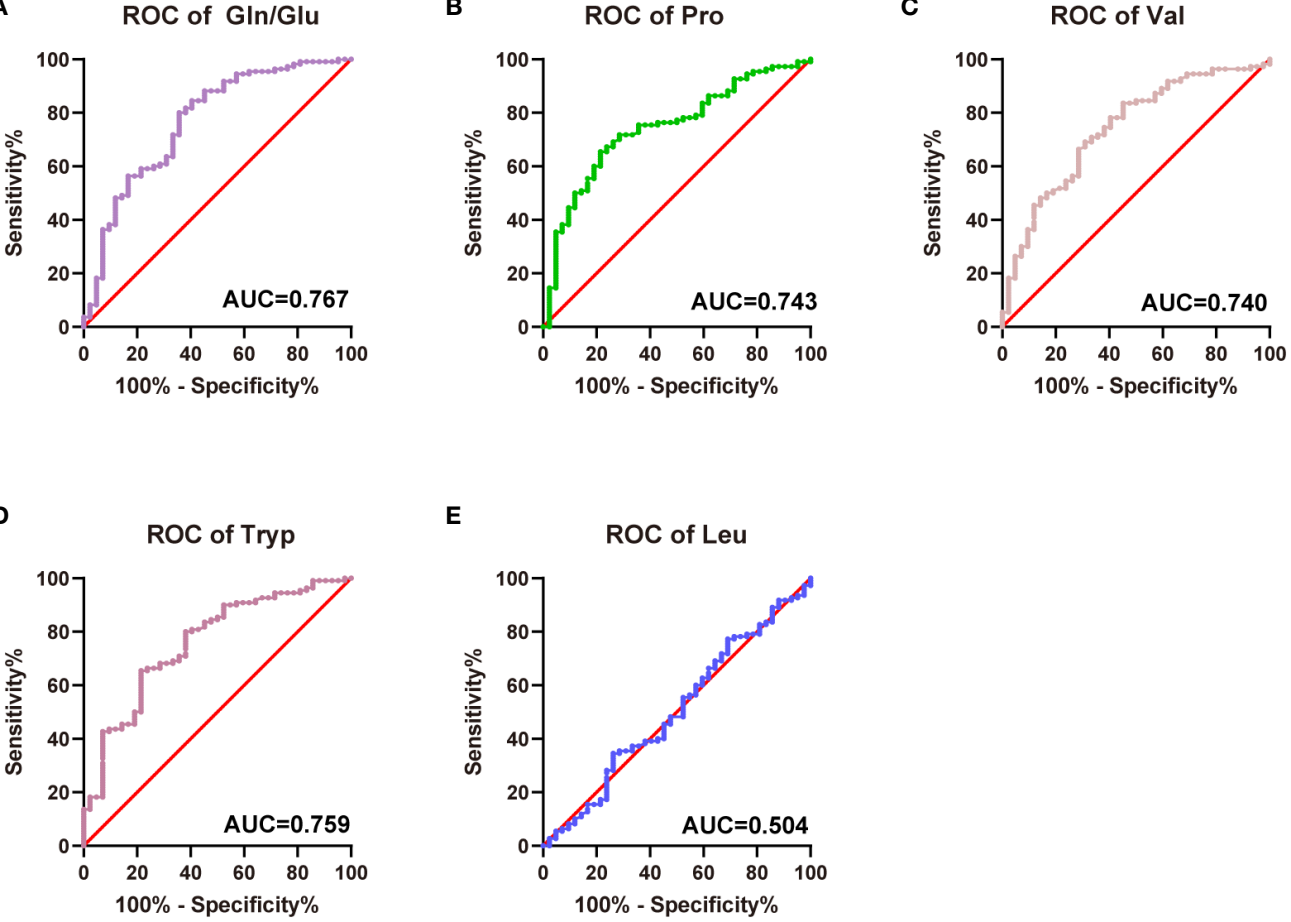
Predictive efficiency and score for the related amino acids in T2D. **(A)** AUC of Gln/Glu ratio in T2D; **(B)** AUC of Pro in T2D; **(C)** AUC of Val in T2D; **(D)** AUC of Tryp in T2D; **(E)** AUC of Leu in T2D.

ROC curves were plotted using Gln/Glu ratio, Pro, Val, Leu, and Tryp to assess the diagnostic values for diabetes, respectively. The Gln/Glu ratio yielded the greatest AUC at 0.767 (0.678-0.856) (**Figure 4A**), followed by Tryp with an AUC of 0.759 (0.673-0.844) (**Figure 4D**). Furthermore, Pro and Val showed AUC values of 0.743 (0.658-0.828) and 0.740 (0.652-0.828), respectively (**Figures 4B, C**). On the contrary, Leu did not demonstrate significant prognostic value (**Figure 4E**). The cut-off value for Gln/Glu ratio was 2.24 (sensitivity 80.0%, specificity 64.9%). For Pro, the cutoff was 3.06 with a sensitivity of 65.45% and a specificity of 78.57%. Val had a cutoff of 1.85, with a sensitivity of 83.6% and a specificity of 54.8%, while Tryp had a cutoff of 3.06, with a sensitivity of 65.45% and a specificity of 35.9%.

### 3.6 Correlation analysis of circulating amino acids and parameters related to diabetes

**Figure 5:**
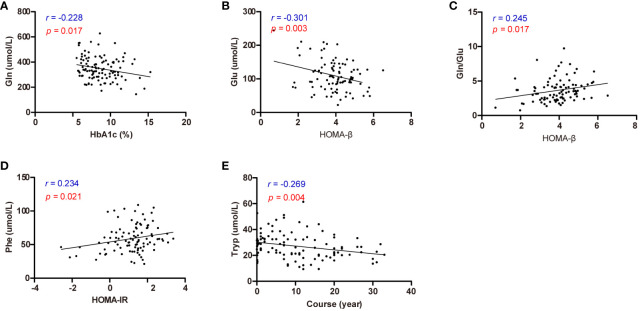
Correlation analysis of effective circulating amino acids with HbA1c, HOMA-β, HOMA-IR, and diabetes course. **(A)** Correlation analysis of Gln with HbA1c. **(B)** Correlation analysis of Glu with HOMA-β. **(C)** Correlation analysis of Gln/Glu with HOMA-β. **(D)** Correlation analysis of Phe with HOMA-IR. **(E)** Correlation analysis of Tryp with diabetes course.

Subsequently, correlation analysis was conducted to explore the relationships between diabetic parameters (HbA1c, HOMA-β, HOMA-IR, and duration) and circulating amino acids in patients diagnosed with T2D (**Supplementary Table S3**). Notably, Gln emerged as the sole amino acid exhibiting a negative correlation with HbA1c (*r* = -0.228, *p* = 0.017) (**Figure 5A**). Moreover, HOMA-β displayed a negative correlation with Glu (*r* = -0.301, *p* = 0.003), while exhibiting a positive correlation with the Gln/Glu ratio (*r* = 0.245, *p* = 0.017) (**Figures 5B, C**). Additionally, HOMA-IR demonstrated a positive correlation with Phe (**Figure 5D**), whereas the duration of diabetes was inversely correlated with Tryp (**Figure 5E**).

### 3.7 Multivariable logistic regression analysis of circulating amino acids and complications in T2D

**Figure 6:**
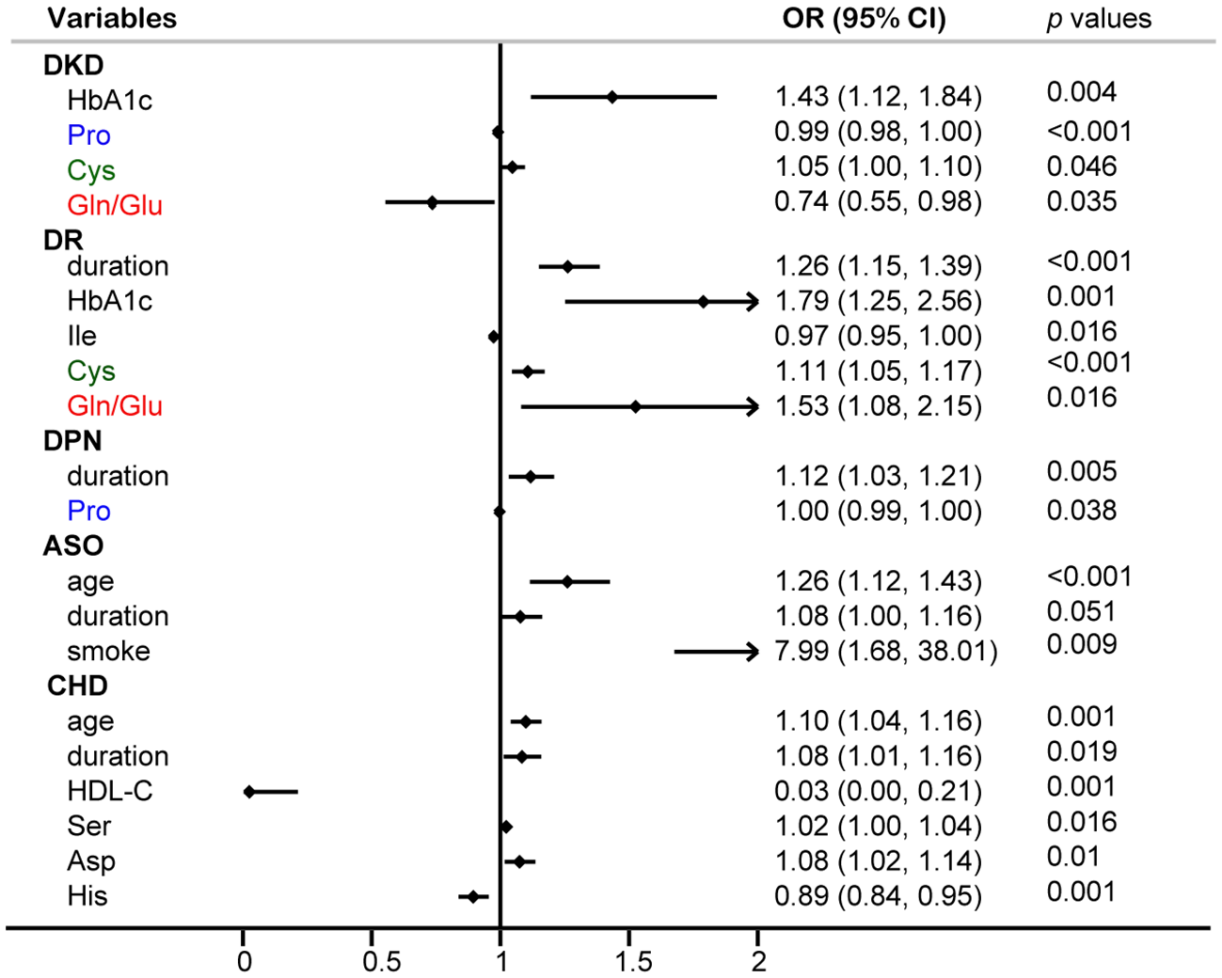
Multivariable logistic regression analysis of circulating amino acids and common diabetic complications.

Logistic regression analysis was performed to identify risk factors for diabetic complications (**Figure 6**). The results identified HbA1c and Cys as independent risk factors for DKD, while Pro and Gln/Glu ratio were recognized as protective factors against DKD. Additionally, the results indicated that disease duration, HbA1c, Cys, and the Gln/Glu ratio were independent risk factors for DR. It is interesting to see that Gln/Glu ratio has different effects on DKD and DR since both DKD and DR are microvascular complications. So, we further separated the patients into four groups: diabetes without DR and DKD, DR without DKD, DKD without DR, and both DKD and DR (**Supplementary Table S4**). Then, we examined the association of Gln/Glu ratio with these distinct patient groups. As compared with the no DKD no DR group, Gln levels in the other three groups were significantly decreased. In addition, Gln levels in DR without DKD were the lowest, but without statistically significance. As for Glu, the trend decreased in the group of DR without DKD, but increased in the groups with DKD (*p* < 0.05). As a result, the Gln/Glu ratio was the highest in DR without DKD group, but with no statistical significance. Disease duration was also singled out as an independent risk factor for DPN. As for ASO, no amino acid was identified as a risk factor, but age, disease duration, and smoking were picked out as significant risk factors.

## 4 Discussion

Prior research has indicated a significant association between T2D and circulating amino acids (18–20).

However, these studies have yielded conflicting results. Furthermore, the majority of these studies did not systematically and comprehensively present the associations. For instance, some studies merely compared amino acid profiles without exploring their correlations with diabetes (18, 19), while others had a limited number of participants (20). Previous studies also identified various amino acids that may be strongly linked to T2D. In this study, we conducted a systematic and comprehensive investigation into the associations between circulating amino acids, conventional diabetic risk factors, and associated complications. We identified the most frequently occurring amino acids from the aforementioned results. Among the examined amino acids, the Gln/Glu ratio exhibited the highest frequency of correlation with metabolic parameters and diabetic complications. These results imply that targeting the Gln/Glu cycle could be a valuable strategy in diabetes prevention.

Circulating amino acids are closely associated with diabetes, obesity, cardiovascular disease, and many other chronic diseases. Previous research has demonstrated associations between circulating BCAAs (29), Gln, Glu (30), and amino acids in the tryptophan-kynurenine pathway (31) with the risk of diabetes and its complications. In the present study, significant alterations were noted in the serum levels of the Gln/Glu ratio, Tryp, Ala, Ser, Pro, Val, Ile, Lys, His, Cys, and Thre.

The correlation analysis revealed that BCAAs and Gln/Glu ratio were the most common amino acids observed in the study. Gln/Glu exhibited negative correlations with BMI, HbA1c, and SUA concentration, while displaying a positive correlation with HDL-C concentration. These findings are consistent with a previous study (32). Furthermore, Gln/Glu ratio was the sole parameter that demonstrated a negative correlation with the TyG index, a marker closely associated with insulin resistance [23] and end-stage kidney disease [30].

Although, several studies have demonstrated the connection between the Gln/Glu ratio and diabetes, however, these studies only focused on the Gln/Glu ratio and diabetic risk. In our current study, we also analyzed the association between Gln/Glu ratio and the related diabetic parameters, including HbA1c, HOMA-β, and HOMA-IR. Furthermore, T2D is a metabolic disease commonly accompanied by different kinds of metabolic dysfunction, such as obesity, hyperuricemia, NAFLD, and so on. In our current research, we also analyzed the role of the Gln/Glu ratio in common metabolic dysfunction. Additionally, the association between serum amino acids and diabetic complications was also analyzed.

The logistic regression analyses revealed that the Gln/Glu ratio serves as an independent risk factor for T2D and obesity. Furthermore, ROC curves supported the prognostic value of the Gln/Glu ratio, Pro, Val, and Leu in relation to T2D, with the Gln/Glu ratio exhibiting the highest AUC. These findings underscore the close correlation between the Gln/Glu ratio and the risk of T2D. Previous studies have demonstrated declined levels of Gln in the adipose tissues of individuals with obesity (17). Notably, prior human studies have linked high circulating levels of Gln and Gln/Glu ratio to a decreased risk of obesity and associated metabolic complications (33, 34). Conversely, lower Gln levels and higher Ala levels have been associated with an increased risk of developing metabolic dysfunction-associated fatty liver disease (35). In the context of our current study, Glu, Val, and Thre were identified as independent risk factors for NAFLD. While our results differ from past findings, it is evident that there is a significant association between Gln and its metabolites with metabolic dysfunction-associated fatty liver disease.

Although numerous studies have affirmed the correlation between diabetes and the Gln/Glu ratio, few have specifically investigated the role of Gln in diabetes management and control. Our results indicate that Gln was the only amino acid displaying a negative correlation with HbA1c in diabetic patients, while Gln/Glu exhibited a positive correlation with HOMA-β. These findings suggest that Gln/Glu may serve as a novel marker for assessing the severity of diabetes. Additionally, HOMA-IR demonstrated a positive correlation with Phe. Further research is necessary to determine whether these amino acids can serve as markers that reflect insulin resistance.

An increasing number of studies have concentrated on investigating the association between serum amino acids and diabetic complications. Factors such as age, diabetes duration, HbA1c levels, BMI, and specific serum amino acids were incorporated in the logistic regression analyses. Various common diabetic complications were examined, including DKD, DR, DPN, CHD, and ASO. Gln/Glu ratio, Pro, and Cys were identified as independent risk factors for DKD. Prior research indicated that serum BCAAs are recognized as a reliable indicator of DKD (7). Surprisingly, none of the BCAAs were found to be significant in our logistic regression analysis. Instead, our findings revealed a strong correlation between Gln/Glu ratio and Pro with both T2D and DKD. It has been reported that the downregulation of P5CS, a rate-limiting enzyme in the Pro biosynthetic pathway, may stimulate *de novo* Gln synthesis (36). This suggests a potential shared mechanism between Gln/Glu ratio and Pro in the context of diabetes and its complications, although further investigation is warranted.

Both DKD and DR are recognized as the most predominant microvascular complications in diabetes. Intriguingly, while Gln/Glu ratio poses a protective factor for DKD, in the case of DR, it appears to act as a risk element. Previous research demonstrated that, Gln metabolism is closely associated with DR. It is reported that, compared with T2DM, Gln and Glu were increased markedly in proliferative DR subjects (37). However, another study showed that, Gln and Glu were significantly increased and decreased in DR subjects, respectively (38). To further explore the connection between Gln and DR, we separated the patients into four groups: diabetes without DR and DKD, DR without DKD, DKD without DR, and both DKD and DR. Gln levels in DR without DKD were the lowest, but without statistically significance. As for Glu, the trend decreased in the group of DR without DKD, but increased in the groups with DKD. As a result, the Gln/Glu ratio was the highest in DR without DKD group, but with no statistically significance. There are some limitations in our study, that the amount of DR patients needed to be increased. Nevertheless, further investigation is essential for a comprehensive understanding of the specific role played by Gln metabolism in DR.

The identification of independent risk factors for DPN revealed that Pro and disease duration play significant roles. While limited studies have investigated the link between amino acids and DPN, a specific study highlighted that sphingolipid metabolism and neuroactive ligand-receptor pathways were implicated in the pathogenesis of DPN through high-throughput sequencing, with multiple amino acid metabolic pathways influencing the onset and progression of DN (39). Past research indicated that the serum Gln/Glu ratio is linked to CHD defined by coronary angiography in Chinese patients (32). However, our current study identified Ser, Asp, and His as independent risk factors for CHD in individuals with diabetes, possibly due to differences in the enrolled populations. In the case of ASO, no amino acids were identified as risk factors, with age, disease duration, and smoking being identified instead. Limited studies have explored the relationship between amino acids and ASO, underscoring the need for further research to elucidate their connection.

## 5 Conclusion

In this study, we investigated the associations between commonly circulating amino acids and traditional diabetic risk factors along with their related complications. Among the examined amino acids, the Gln/Glu ratio exhibited the highest frequency of correlation with metabolic parameters and diabetic complications. Our findings shed light on the crucial role of Gln metabolism in T2D and suggest a potential novel target for strategies aimed at preventing diabetes.

## Data availability statement

The raw data supporting the conclusions of this article will be made available by the authors, without undue reservation.

## Ethics statement

The studies involving humans were approved by Ethics Review Committee of Chu Hsien-I Memorial Hospital of Tianjin Medical University (No. DXBYYkMEC2021-14). The studies were conducted in accordance with the local legislation and institutional requirements. The ethics committee/institutional review board waived the requirement of written informed consent for participation from the participants or the participants' legal guardians/next of kin because the biospecimens were obtained from previous clinical diagnosis, and the waiver of informed consent does not adversely affect the rights and health of subjects.

## Author contributions

FH: Conceptualization, Formal Analysis, Funding acquisition, Investigation, Writing – original draft, Writing – review & editing. CX: Conceptualization, Data curation, Methodology, Writing – review & editing. XH: Formal Analysis, Methodology, Software, Writing – review & editing. YL: Data curation, Investigation, Methodology, Writing – review & editing. YZ: Formal Analysis, Supervision, Visualization, Writing – original draft. BS: Data curation, Formal Analysis, Supervision, Validation, Visualization, Writing – review & editing. LC: Funding acquisition, Supervision, Validation, Visualization, Writing – review & editing.
