# Supplementary material for: Circulating glutamine/glutamate ratio is closely associated with type 2 diabetes and its associated complications
Source: Front Endocrinol (Lausanne). 2024 Jul 18;15:1422674. doi: 10.3389/fendo.2024.1422674 (PMC11291334; doi:10.3389/fendo.2024.1422674)
Supplement: Supplementary file 1 [file Table_1.docx]

**sTable1 Concentrations of circulating amino acid in patients with T2D and healthy controls**

|  | **NC (n=42)** | **T2D (n=110)** | ***P*-values** |
| --- | --- | --- | --- |
| **Gln** |  |  |  |
| Mean (SD) | 380.48 ± 74.17 | 347.83 ± 92.47* | 0.042 |
| Median [Min, Max] | 372.16 [243.63, 532.69] | 331.9852 [144.39, 627.87] |  |
| **Glu** |  |  |  |
| Mean (SD) | 77.95 ± 35.56 | 109.87 ± 43.3884*** | <0.001 |
| Median [Min, Max] | 71.82 [33.66, 190.71] | 104.47 [22.31, 244.0] |  |
| **Gln/Glu** |  |  |  |
| Mean (SD) | 5.77 ± 2.36 | 3.64 ± 1.61*** | <0.001 |
| Median [Min, Max] | 5.68 [1.56, 11.22] | 3.43 [0.75, 9.73] |  |
| **Ala** |  |  |  |
| Mean (SD) | 220.40 ± 65.11 | 270.47 ± 87.13** | 0.001 |
| Median [Min, Max] | 201.5 [118.9, 397.1] | 257.25 [64.12, 518.0] |  |
| **Ser** |  |  |  |
| Mean (SD) | 100.29 ± 35.18 | 140.51 ± 48.97*** | <0.001 |
| Median [Min, Max] | 90.63 [55.05, 225.7] | 134.05 [47.49, 297.5] |  |
| **Pro** |  |  |  |
| Mean (SD) | 113.52 ± 110.45 | 171.84 ± 99.52*** | <0.001 |
| Median [Min, Max] | 93.38 [50.75, 758.3] | 133.10 [16.55, 441.5] |  |
| **Val** |  |  |  |
| Mean (SD) | 201.80 ± 68.72 | 268.09 ± 81.78*** | <0.001 |
| Median [Min, Max] | 183.40 [105.8, 402.3] | 263.65 [80.72, 510.6] |  |
| **Leu** |  |  |  |
| Mean (SD) | 176.99 ± 56.46 | 177.87 ± 57.67 | 0.933 |
| Median [Min, Max] | 165.50 [70.83, 314.3] | 168.10 [77.13, 362.0] |  |
| **Ile** |  |  |  |
| Mean (SD) | 77.10 ± 22.69 | 92.05 ± 32.95** | 0.008 |
| Median [Min, Max] | 73.26 [41.08, 128.4] | 86.24 [32.04, 182.0] |  |
| **Asp** |  |  |  |
| Mean (SD) | 21.70 ± 9.13 | 23.51 ± 10.03 | 0.168 |
| Median [Min, Max] | 21.88 [8.20, 60.37] | 23.49 [0.25, 50.92] |  |
| **Lys** |  |  |  |
| Mean (SD) | 107.30 ± 32.20 | 139.05 ± 40.40*** | <0.001 |
| Median [Min, Max] | 102.45 [68.05, 197.40] | 131.90 [61.13, 243.0] |  |
| **Met** |  |  |  |
| Mean (SD) | 26.78 ± 5.55 | 28.35 ± 7.95 | 0.243 |
| Median [Min, Max] | 25.90 [17.83, 36.76] | 26.75 [12.76, 58.27] |  |
| **His** |  |  |  |
| Mean (SD) | 61.41 ± 13.68 | 70.09 ±15.57** | 0.001 |
| Median [Min, Max] | 59.12 [36.48, 94.85] | 68.81 [25.65, 108.5] |  |
| **Tryp** |  |  |  |
| Mean (SD) | 37.50 ± 11.60 | 26.98 ± 9.90*** | <0.001 |
| Median [Min, Max] | 36.37 [16.55, 67.08] | 25.54 [9.38, 61.45] |  |
| **Phe** |  |  |  |
| Mean (SD) | 61.09 ± 17.55 | 58.45 ± 56.25 | 0.445 |
| Median [Min, Max] | 59.76 [29.77, 100.40] | 56.25 [21.19, 109.10] |  |
| **Cys** |  |  |  |
| Mean (SD) | 22.35 ± 9.11 | 27.86 ± 12.96* | 0.013 |
| Median [Min, Max] | 20.22 [8.19, 48.09] | 26.42 [6.22, 65.07] |  |
| **Thre** |  |  |  |
| Mean (SD) | 79.82 ± 21.44 | 95.17 ± 446.01* | 0.036 |
| Median [Min, Max] | 75.51 [52.6, 135.2] | 88.96 [19.65, 375.6] |  |

* means compared with the control group, *p* < 0.05; ** means compared with the control group, *p* < 0.01; *** means compared with the control group, *p* < 0.001.
