# Supplementary material for: Circulating glutamine/glutamate ratio is closely associated with type 2 diabetes and its associated complications
Source: Front Endocrinol (Lausanne). 2024 Jul 18;15:1422674. doi: 10.3389/fendo.2024.1422674 (PMC11291334; doi:10.3389/fendo.2024.1422674)
Supplement: Supplementary file 2 [file Table_2.docx]

sTable2 Correlation analysis of circulating amino acid and metabolic indicators in patients with T2D and healthy controls

|  | Gln | Glu | Gln/Glu | Ala | Ser | Pro | Val | Leu | Ile | Asp | Lys | Met | His | Tryp | Phe | Cys | Thre |
| --- | --- | --- | --- | --- | --- | --- | --- | --- | --- | --- | --- | --- | --- | --- | --- | --- | --- |
| BMI |  |  |  |  |  |  |  |  |  |  |  |  |  |  |  |  |  |
| *r* | -0.083 | 0.219^**^ | -0.328^**^ | 0.152 | 0.032 | 0.037 | 0.261^**^ | 0.299^**^ | 0.231^**^ | 0.029 | 0.205^*^ | 0.123 | 0.032 | 0.085 | 0.255^**^ | -0.004 | 0.022 |
| *p* | 0.308 | **0.007** | **< 0.001** | 0.062 | 0.698 | 0.655 | **0.001** | **< 0.001** | **0.004** | 0.720 | **0.011** | 0.131 | 0.697 | 0.297 | **0.002** | 0.962 | 0.787 |
| HbA1c |  |  |  |  |  |  |  |  |  |  |  |  |  |  |  |  |  |
| *r* | -0.274^**^ | 0.284^**^ | -0.380^**^ | 0.243^**^ | 0.292^**^ | 0.225^**^ | 0.293^**^ | 0.008 | 0.241^**^ | 0.053 | 0.248^**^ | 0.043 | 0.159 | -0.278^**^ | -0.100 | 0.058 | 0.044 |
| *p* | **0.001** | **< 0.001** | **< 0.001** | **0.003** | **<0.001** | **0.005** | **< 0.001** | 0.924 | **0.003** | 0.515 | **0.002** | 0.603 | 0.050 | **0.001** | 0.222 | 0.474 | 0.593 |
| SUA |  |  |  |  |  |  |  |  |  |  |  |  |  |  |  |  |  |
| *r* | 0.016 | 0.078 | -0.161^*^ | 0.103 | 0-.029 | -0.033 | 0.095 | 0.119 | 0.016 | 0.140 | 0.024 | -0.083 | -0.007 | 0.033 | 0.147 | -0.078 | -0.069 |
| *p* | 0.848 | 0.337 | **0.048** | 0.207 | 0.725 | 0.691 | 0.247 | 0.145 | 0.844 | 0.084 | 0.766 | 0.311 | 0.931 | 0.683 | 0.070 | 0.337 | 0.401 |
| TG |  |  |  |  |  |  |  |  |  |  |  |  |  |  |  |  |  |
| *r* | -0.071 | -0.072 | -0.041 | 0.074 | 0.039 | -0.033 | 0.300^**^ | 0.268^**^ | 0.187^*^ | 0.199^*^ | 0.102 | -0.023 | 0.016 | 0.095 | 0.207^*^ | -0.046 | -0.070 |
| *p* | 0.382 | 0.380 | 0.620 | 0.363 | 0.630 | 0.683 | **< 0.001** | **0.001** | **0.021** | **0.014** | 0.213 | 0.774 | 0.846 | 0.243 | **0.011** | 0.570 | 0.389 |
| TC |  |  |  |  |  |  |  |  |  |  |  |  |  |  |  |  |  |
| *r* | 0.043 | -0.108 | 0.027 | -0.070 | 0.020 | -0.156 | 0.057 | 0.041 | 0.060 | 0.210^**^ | -0.003 | -0.141 | -0.112 | -0.036 | 0.061 | -0.065 | -0.104 |
| *p* | 0.597 | 0.183 | 0.746 | 0.391 | 0.811 | 0.055 | 0.489 | 0.612 | 0.461 | **0.010** | 0.970 | 0.082 | 0.168 | 0.663 | 0.452 | 0.426 | 0.204 |
| HDL-c |  |  |  |  |  |  |  |  |  |  |  |  |  |  |  |  |  |
| *r* | 0.043 | -0.100 | 0.173^*^ | -0.261^**^ | -0.144 | -0.208^*^ | -0.328^**^ | -0.190^*^ | -0.217^**^ | -0.152 | -0.274^**^ | -0.178^*^ | -0.227^**^ | -0.068 | -0.255^**^ | -0.110 | -0.044 |
| *p* | 0.604 | 0.224 | **0.033** | **0.001** | 0.078 | **0.010** | **< 0.001** | **0.019** | **0.007** | 0.062 | **0.001** | **0.029** | **0.005** | 0.410 | **0.002** | 0.177 | 0.591 |
| LDL-c |  |  |  |  |  |  |  |  |  |  |  |  |  |  |  |  |  |
| *r* | 0.108 | -0.059 | -0.007 | -0.086 | -0.009 | -0.126 | -0.010 | -0.019 | 0.028 | 0.162^*^ | -0.053 | -0.147 | -0.110 | -0.043 | 0.052 | -0.045 | -0.096 |
| *p* | 0.186 | 0.473 | 0.934 | 0.292 | 0.912 | 0.122 | 0.901 | 0.821 | 0.735 | **0.047** | 0.516 | 0.071 | 0.178 | 0.596 | 0.527 | 0.581 | 0.240 |
| TyG |  |  |  |  |  |  |  |  |  |  |  |  |  |  |  |  |  |
| *r* | -0.095 | 0.114 | -0.250^**^ | 0.097 | -0.003 | -0.046 | 0.275^**^ | 0.256^**^ | 0.173^*^ | 0.165^*^ | 0.075 | -0.058 | -0.011 | 0.059 | 0.177^*^ | -0.073 | -0.152 |
| *p* | 0.246 | 0.163 | **0.002** | 0.235 | 0.970 | 0.576 | **0.001** | **0.001** | **0.033** | **0.043** | 0.356 | 0.476 | 0.891 | 0.470 | **0.029** | 0.373 | 0.061 |

* means compared with the control group, *p* < 0.05; ** means compared with the control group, *p* < 0.01; The bold values denote statistical significance at *P* < 0.05 level.
