# Supplementary material for: Circulating glutamine/glutamate ratio is closely associated with type 2 diabetes and its associated complications
Source: Front Endocrinol (Lausanne). 2024 Jul 18;15:1422674. doi: 10.3389/fendo.2024.1422674 (PMC11291334; doi:10.3389/fendo.2024.1422674)
Supplement: Supplementary file 3 [file Table_3.docx]

sTable 3 Correlation analysis of circulating amino acids and parameters related to diabetes

|  | Gln | Glu | Gln/Glu | Ala | Ser | Pro | Val | Leu | Ile | Asp | Lys | Met | His | Tryp | Phe | Cys | Thre |
| --- | --- | --- | --- | --- | --- | --- | --- | --- | --- | --- | --- | --- | --- | --- | --- | --- | --- |
| HbA1c |  |  |  |  |  |  |  |  |  |  |  |  |  |  |  |  |  |
| *r* | **-0.228*** | 0.087 | -0.116 | 0.085 | 0.074 | 0.091 | 0.081 | -0.017 | 0.133 | -0.022 | 0.025 | -0.030 | -0.013 | -0.057 | -0.122 | -0.104 | -0.090 |
| *p* | **0.017** | 0.366 | 0.229 | 0.376 | 0.440 | 0.345 | 0.402 | 0.859 | 0.166 | 0.822 | 0.793 | 0.753 | 0.889 | 0.555 | 0.204 | 0.281 | 0.352 |
| HOMA-β |  |  |  |  |  |  |  |  |  |  |  |  |  |  |  |  |  |
| *r* | 0.152 | **-0.301** | **0.245^*^** | 0.155 | -0.003 | -0.001 | 0.010 | 0.048 | 0.011 | 0.125 | 0.093 | 0.118 | 0.121 | 0.148 | 0.152 | -0.086 | 0.097 |
| *p* | 0.142 | **0.003^**^** | **0.017** | 0.134 | 0.980 | 0.995 | 0.925 | 0.647 | 0.913 | 0.229 | 0.372 | 0.255 | 0.244 | 0.153 | 0.142 | 0.409 | 0.352 |
| HOMA-IR |  |  |  |  |  |  |  |  |  |  |  |  |  |  |  |  |  |
| *r* | 0.193 | -0.192 | 0.191 | 0.026 | -0.006 | -0.180 | 0.096 | 0.140 | -0.043 | 0.182 | 0.019 | 0.093 | 0.020 | 0.161 | **0.234^*^** | -0.031 | -0.058 |
| *p* | 0.059 | 0.059 | 0.062 | 0.800 | 0.953 | 0.078 | 0.350 | 0.172 | 0.674 | 0.074 | 0.856 | 0.366 | 0.846 | 0.115 | **0.021** | 0.765 | 0.573 |
| Duration |  |  |  |  |  |  |  |  |  |  |  |  |  |  |  |  |  |
| *r* | -0.123 | 0.117 | -0.158 | 0.098 | -0.006 | 0.093 | -0.120 | -0.186 | -0.068 | -0.146 | -0.008 | 0.030 | 0.069 | **-0.269** | -0.135 | 0.047 | 0.118 |
| *p* | 0.199 | 0.222 | 0.100 | 0.309 | 0.952 | 0.333 | 0.210 | 0.051 | 0.482 | 0.127 | 0.933 | 0.752 | 0.473 | **0.004^**^** | 0.160 | 0.628 | 0.220 |

* means compared with the control group, *p* < 0.05; ** means compared with the control group, *p* < 0.01; The bold values denote statistical significance at *P* < 0.05 level.
