# Supplementary material for: Circulating glutamine/glutamate ratio is closely associated with type 2 diabetes and its associated complications
Source: Front Endocrinol (Lausanne). 2024 Jul 18;15:1422674. doi: 10.3389/fendo.2024.1422674 (PMC11291334; doi:10.3389/fendo.2024.1422674)
Supplement: Supplementary file 4 [file Table_4.docx]

sTable 4 Circulating levels of Gln and Glu in DR and DKD patients

|  | **Diabetes without DR and DKD (n = 34)** | **DR without DKD**  **(n = 9)** | **DKD without DR**  **(n = 39)** | **DKD + DR**  **(n = 37)** |
| --- | --- | --- | --- | --- |
| **Glutamine** | 388.0 ± 106.0 | 314.2 ± 78.0^*^ | 331.9 ± 76.6^*^ | 332.0 ± 85.0^*^ |
| **Glutamate** | 109.3 ± 31.8 | 80.4 ± 36.4 | 113.1 ± 44.7^#^ | 115.0 ± 51.2^#^ |
| **Gln/Glu** | 3.84 ± 1.47 | 4.55 ± 1.72 | 3.42 ± 1.65 | 3.42 ± 1.63 |

* means compared with the diabetes without DR and DKD group, *p* < 0.05;

^#^ means compared with the DR without DKD group, *p* < 0.05;
